# Humidified and Heated Cascade Impactor for Aerosol Sizing

**DOI:** 10.3389/fbioe.2020.589782

**Published:** 2020-11-13

**Authors:** Caroline Majoral, Allan L. Coates, Alain Le Pape, Laurent Vecellio

**Affiliations:** ^1^INSERM, Research Center for Respiratory Diseases, Tours, France; ^2^Université de Tours, Tours, France; ^3^Hospital for Sick Children, Toronto, ON, Canada

**Keywords:** aerosol, cascade impactor, particle, inhalation, size, temperature, humidity

## Abstract

Aerosol sizing is generally measured at ambient air but human airways have different temperature (37°C) and relative humidity (100%) which can affect particle size in airways and consequently deposition prediction. This work aimed to develop and evaluate a new method using cascade impactor to measure particle size at human physiological temperature and humidity (HPTH) taking into account ambient air conditions. A heated and humidified trachea was built and a cascade impactor was heated to 37°C and humidified inside. Four medical aerosols [jet nebulizer, mesh nebulizer, Presurized Metered Dose Inhaler (pMDI), and Dry Powder Inhaler (DPI)] under ambient conditions and at HPTH were tested. MMAD was lower at HPTH for the two nebulizers; it was similar at ambient conditions and HPTH for pMDI, and the mass of particles smaller than 5 μm decreased for DPI at HPTH (51.9 vs. 82.8 μg/puff). In conclusion, we developed a new method to measure particle size at HPTH affecting deposition prediction with relevance. *In vivo* studies are required to evaluate the interest of this new model to improve the precision of deposition prediction.

## Introduction

Aerosol particle size is a key parameter for predicting aerosol deposition in the airways of the lungs. Several methods of particle sizing can be used, cascade impactors and lasers being the most common. Cascade impactors offer the major advantage of measuring the aerodynamic diameter of the drug studied.

They are used in different regulatories (ISO 27427, 2012) and pharmacopeias (USP 28-Nf 23, 2005) with a continuous and constant air flow. However, this experimental set up does not take into account the effect of ventilation parameters from the patient which can modify the particle size. Sangwan et al. (2003) has used a low flow cascade impactor (1 L/min) with a breathing machine to mimic patient inhalation. They used clinically relevant breathing patterns to simulate aerosol delivery on the bench recognizing that, during a drug treatment the patient would be breathing a mixture of air at a certain tidal volume and frequency as well as air from the nebulizer and that this degree of ambiant air mixing might influence the final inhaled distribution. Finally in a series of human studies demonstrated the relationship between predicting deposition via bench studies and effects on actual deposition measure particle size at different ventilation conditions. The low flow cascade impactor has the advantage to reduce the impact of air sampling on ventilation parameters and to reduce the particle evaporation effect in standing cloud set up (Solomita and Smaldone, 2009) but it has the unconvenient to collect a small fraction of aerosol which can be different to the aerosol cloud (Vecellio None et al., 2001).

*In vitro* measurements of aerosol particle size with a cascade impactor are usually made at ambient conditions of temperature and relative humidity (RH).

It has been shown that the temperature of a jet nebulizer outlet can decrease by more than 10°C, leading to evaporation of the droplets when entering the cascade impactor placed at ambient temperature. Previous studies have investigated the effect of temperature on aerosol sizing by cooling the impactor in order to limit droplet evaporation (Stapleton and Finlay, 1998; Kwong et al., 2000; Zhou et al., 2005a; Rao et al., 2010). Several studies have also investigated the effect of ambient air humidity on particle size distribution by sizing the aerosols at several different values of relative humidity (Prokop et al., 1995; Finlay et al., 1997; Nerbrink et al., 2003; Zhou et al., 2005b). However, the human respiratory tract is at 37°C and almost 100%RH (Ferron, 1977; Ferron et al., 1985; O’Doherty and Miller, 1993), which can alter particle size either through evaporation or condensation (i.e., hygroscopic growth) and can therefore affect the relevance of the prediction of deposition in the respiratory tract.

Studies have focused on mathematically modeling heat and water transport in the human respiratory tract (Daviskas et al., 1990; Eisner, 1992; Finlay and Stapleton, 1995; Zhang et al., 2006), or on establishing equations to predict the change in size of hygroscopic inhaled particles (Ferron, 1977; Ferron et al., 1988; Gradon and Podgórski, 1996; Broday and Georgopoulos, 2001; Longest and Hindle, 2012; Boudin et al., 2020). Particle growing in airways has been recently considered as a potential interest to reduce upper airways deposition and increase lung penetration, and deposition when using submicronic aerosols (Longest and Hindle, 2012; Spence et al., 2019). Martin et al. (1988) developed a model for particle sizing composed of a trachea and a cascade impactor at 37°C and either 30%RH or 97.5%RH. However, their model did not duplicate what happens *in vivo*. Instead of humidifying the aerosol after its generation, they humidified and heated the ambient air to 37°C before it entered the model. In fact, when a patient inhales an aerosol, the air he/she breathes comes from the atmosphere and is at ambient conditions of temperature, and humidity. In another study, Bell and Ho (1981) developed an experimental system which mixed a dry monodisperse aerosol with water-saturated air at 37°C at the top of a growth tube. They sized aerosol particles at the exit of this tube using an optical particle counter, thus measuring the geometric diameter, but neither the aerodynamic diameter nor the drug mass contained in the particles.

Consequently, temperature and humidity affect particle size. Particle size modification in airways depends on device and formulation. Different mathematical models have been developed to predict this size modification but they are complex to use. Different experimental measurement methods have been developed to measure *in vitro* aerosol size at 37°C and 100% but they have not measured the size of aerosol drug in realistic ambiant air mixing conditions, i.e., with ambiant temperature and humidity crossing the device and followed by a heating and humidifying just after aerosol delivery.

The purpose of the present study was (1) to develop an *in vitro* cascade impactor measurement method approaching human temperature and humidity with the respect of the ambient air before penetration in the trachea/induction port model, (2) to compare aerosol particle size measured at ambient conditions and at HPTH to evaluate the relevance of our method.

## Materials and Methods

### Materials

#### Experimental Set Up at Ambient Air

**FIGURE 1:**
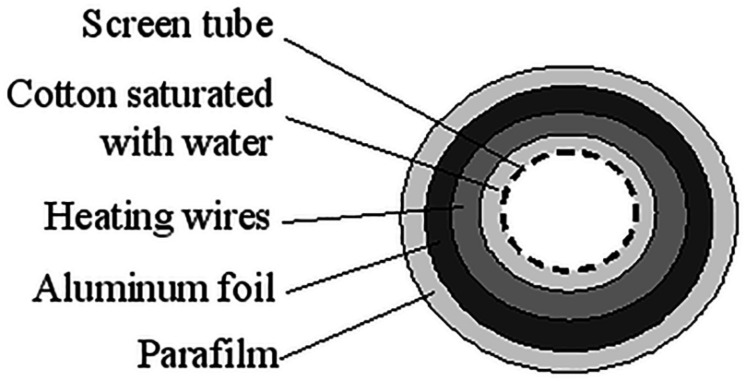
Cross-section of the new trachea.

**FIGURE 2:**
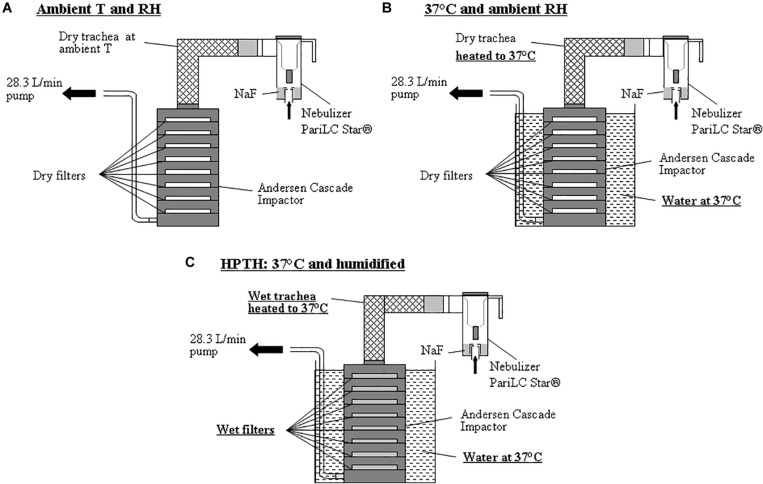
Experimental set-ups using ACI sampling at 28.3 L/min with stage’s substrates (filters) for 3 different conditions: **(A)** Aerosol measurement using cascade impactor at ambient temperature and ambient relative humidity. **(B)** Aerosol measurement using cascade impactor in a bath of water at 37°C and ambient relative humidity. **(C)** Aerosol measurement using cascade impactor in a bath of water at 37°C and humidified air inside the trachea model and the cascade impactor using the new trachea and wetted stage’s substrates (HPTH: human physiological temperature and humidity).

The aerosol were sized using an 8-stage Andersen Cascade Impactor (ACI) (Mark II, Ecomesure, France) operating at 28.3 L/min for nebulizers and pMDI, and operating at 60 L/min for DPI. An artificial trachea for the ACI was created using a coarse screen wrapped with cotton with a heating coil running inside the walls which were then covered with aluminum foil and plastic (Figure 1). Absorbent glass fiber filters (Type A/E Glass, Pall Corporation, United States) were used on each plate of the impactor. The trachea was kept dry and not heated, and the cascade impactor was used with dry filters and at ambient temperature (22 ± 3°C; 50 ± 10%RH) (Figure 2A).

#### Experimental Set Up at 37°C and Ambient RH

Same cascade impactor and trachea as described above were used. The trachea was dry and heated to 37°C and the impactor was warmed in a water bath at 37°C. A potentiometer was used to regulate the temperature of the heating coil to 37°C and a temperature sensor was used to measure the temperature of the trachea and of the water bath. The filters on cascade impactor stages were dry so that humidity inside the impactor was the same as that of the ambient air, as the impactor was perfectly watertight (Figure 2B).

#### Experimental Set Up at 37°C and 100% RH

Same cascade impactor and trachea as described above were used. In this experimental set up, the trachea was humidified inside with water and heated to 37°C, and the cascade impactor filters were moistened with 3 mL of water and placed in a water bath at 37°C (Figure 2C).

#### Inhalation Drug Delivery Devices

Different inhalation drug were tested: Jet nebulizer (PariLC Star^®^ with Turbo Boy N^®^ compressor, Pari, Germany) filled with 2 mL of terbutaline (Bricanyl^®^ 5 mg/2 mL, Astra Zeneca, France) or with 2 mL of 1%w/v NaF solution, a vibrating mesh nebulizer (Aeroneb Go^®^, Ireland, United States) filled with 2 mL of terbutaline (Bricanyl^®^ 5 mg/2 mL, Astra Zeneca, France), a salbutamol Presurized Metered Dose Inhaler (PMDI) (Ventoline^®^ 100 μg/puff, GlaxoSmithKline, France), and a terbutaline Dry Powder Inhaler (DPI) (Bricanyl^®^ Turbuhaler^®^ 500 μg/puff, Astra Zeneca, France). Only the Pari LC Star^®^ nebulizer with NaF was measured at 37°C-ambiant humidity. Others inhalation drug delivery device were sized at ambient conditions (Figure 2A) and at HPTH (Figure 2C).

### Methods

#### Experimental Setup Validation

Three milliliter of water were poured onto dry absorbent filters (Type A/E Glass, Pall Corporation, United States) laid on each impactor plate and they were each immediately weighed. The experimental set-up at HPTH described in Figure 2C was then carried out, substituting the nebulizer by an absolute filter to limit the risk of air particle contamination on wet filters. The impactor pump, operating at 28.3 L/min, was turned on for 5 min. The impactor was then dismantled and the filters were weighed again. The difference between the weights of the filters before and after the 5 min of sampling corresponded to the quantity of water evaporated inside the impactor during the 5 min. The experiment was performed three times. The relative humidity inside the trachea was measured after its humidification by a humidity measuring stick (Testo, France). Each experiment was carried out in triplicate.

#### Aerosol Sizing

For the two nebulizers, nebulization was stopped when no more aerosol was produced. After Naf nebulization, each filter and impactor stage were placed in 10 mL of 25% TISAB solution (TISAB IV, Riedel-de Haën, Germany) and was assayed with a fluoride electrode.

After terbutaline nebulization, each filter was placed in a centrifuge tube and each impactor stage was placed in 20 mL of sodium hydroxide 0.1M. The amount of drug was assayed by UV-spectrophotometry (Spectronic Unicam, Helios, United Kingdom). Residual drug mass in nebulizers was measured by drug assay method.

For pMDI, a total of 30 puffs of the were delivered for each experiment. The UV spectrophotometer was calibrated to measure salbutamol, and the filters were processed as described above for the nebulizers.

Terbutaline Turbuhaler^®^ DPI required a 60 L/min pump instead of a 28.3 L/min pump. As the ACI was calibrated at 28.3 L/min, the usual values given for the cut-off diameters were only valid for the impactor operating at 28.3 L/min. The cut-off diameters were determined for a 60 L/min flow rate according to the following formula (Van Oort et al., 1996; Marple et al., 2003):

$${\text{D}}_{60\,L/\min}={\text{D}}_{28.3\,L/\min}\,\sqrt{\frac{28.3}{60}}$$

where D_6__0L__/min_ and D_28_._3L__/min_ correspond to the cut-off diameters for the ACI operating at 60 and 28.3 L/min respectively.

The values of the cut-off diameters for a flow rate of 28.3 L/min were 9, 5.8, 4.7, 3.3, 2.1, 1.1, 0.7, and 0.4 μm for stages 0–7, respectively. The values of the cut-off diameters for a flow rate of 60 L/min were 6.2, 4.0, 3.2, 2.3, 1.4, 0.8, 0.5, and 0.3 μm for stages 0–7, respectively.

A total of 20 puffs of terbutaline Turbuhaler^®^ DPI were generated for each experiment. The filters were processed as described above for the nebulizers.

### Analysis of the Results

NaF aerosol particle size distributions were represented as mass deposited per stages in the cascade impactor. The percentage of recovery from the impactor and the trachea was expressed from the nebulizer load for the nebulizers, the difference from 100% corresponding to the residual volume. The deposition in the trachea was calculated by subtracting (total deposition on the impactor stages + residual volume) from the initial charge for nebulizers, and (total deposition on the impactor stages) from the emitted dose for the PMDI and DPI.

The results were also expressed in terms of Mass Median Aerodynamic Diameter (MMAD), total deposited mass on the stages of the impactor, % recovery from the impactor and the trachea of the nebulizer load for the nebulizers and of the emitted dose for the PMDI and DPI, and mass of particles smaller than 5 μm, also called the respirable mass or fine particle dose and predicting deposited mass in the lungs (Coates et al., 1998; ISO 27427, 2012).

## Results

### Validation of the Method

A volume of 0.41 ± 0.07 mL of water was evaporated from each filter during the 5 min of sampling. There was no difference between each stage in term of evaporated volume water. These results validated the humidification of the air inside the impactor. The relative humidity inside the trachea after its humidification was superior to 95%, which corresponds to the relative humidity inside the respiratory tract.

### Aerosol Sizing at Different Air Temperature and Humidity Conditions

MMAD, total deposited mass on the stages of the ACI and % recovery from the impactor and the trachea of the nebulizer load of 1%w/v NaF aerosol nebulized with PariLC Star^®^ for the three experimental set-ups are summarized in Table 1. MMAD equaled 2.6 ± 0.2, 1.3 ± 0.1, and 1.8 ± 0.1 μm for ambient T and RH, 37°C and ambient RH, and HPTH respectively. Total deposited mass on the stages of the impactor was similar for the three experimental set-ups (10.0 ± 0.7, 9.2 ± 0.3, and 9.3 ± 0.7 mg).

**TABLE 1:** MMAD, total deposited mass on the stages of the ACI and % recovery from the impactor and the trachea of the nebulizer load of 1%w/v NaF aerosol nebulized with PariLC Star^®^ for the three experimental set-ups [Ambient T and RH; 37°C and ambient RH; 37°C and humidified (HPTH: human physiological temperature and humidity)], expressed as mean ± standard deviation.

	**MMAD (μm)**	**Total deposited mass on the stages of the ACI (mg) (% of the nebulizer load)**	**% recovery from the trachea of the nebulizer load**
(1) Ambient T and RH	2.6 ± 0.2	10.0 ± 0.7 (47 ± 3%)	4 ± 4%
(2) 37°C and ambient RH	1.3 ± 0.1	9.2 ± 0.3 (46 ± 2%)	1 ± 2%
(3) HPTH	1.8 ± 0.1	9.3 ± 0.7 (43 ± 3%)	3 ± 3%

**FIGURE 3:**
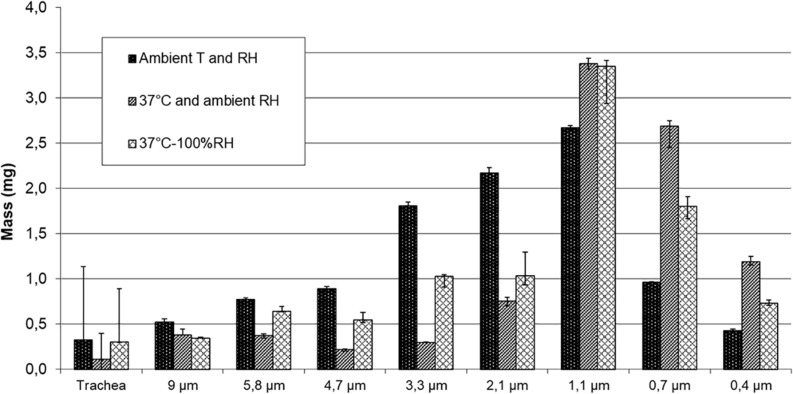
Deposited mass on impactor stages using 1%w/v NaF aerosol nebulized with PariLC Star^®^ for the three conditions of temperature (T) and relative humidity (RH): ambient T and ambient RH; 37°C and ambient RH; 37°C and 100% RH inside the trachea model and the cascade impactor.

Figure 3 shows the deposited mass on impactor stages with NaF aerosol nebulized by PariLC Star^®^ for the three experimental set-ups.

**FIGURE 4:**
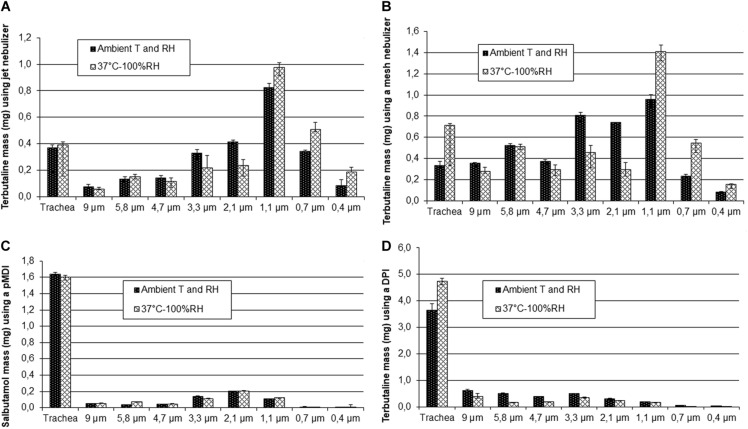
Deposited mass on impactor stages for both ambient conditions and 37°C humidified (HPTH: human physiological temperature and humidity) using the Pari LC Star^®^ jet nebulizer **(A)**, the Aeroneb Go^®^ mesh nebulizer **(B)**, 30 puffs of Ventoline^®^ pMDI **(C)** and 20 puffs of Bricanyl^®^ Turbuhaler^®^ DPI **(D)**.

MMAD, total deposited mass on the stages of the ACI, % recovery from the impactor and the trachea of the nebulizer load for the nebulizers and of the emitted dose for the PMDI and DPI, and mass of particles smaller than 5 μm of terbutaline nebulized with PariLC Star^®^ and Aeroneb Go^®^ nebulizers, salbutamol PMDI and terbutaline Turbuhaler^®^ DPI for both ambient conditions and HPTH are summarized in Table 2. MMAD values at ambient conditions and HPTH were, respectively, 2.1 ± 0.3 and 1.6 ± 0.1 μm for PariLC Star^®^, 3.3 ± 0.1 and 2.0 ± 0.2 μm for Aeroneb Go^®^, 3.1 ± 0.0 and 3.1 ± 0.1 μm for salbutamol PMDI, 3.7 ± 0.0 and 3.2 ± 0.1 μm for terbutaline Turbuhaler^®^ DPI. Total deposited mass on the stages of the impactor was similar at ambient conditions and HPTH for all the devices except for terbutaline Turbuhaler^®^ DPI: it was, respectively, 2.3 ± 0.2 and 2.5 ± 0.3 mg for PariLC Star^®^, 3.9 ± 0.3 and 3.9 ± 0.3 mg for Aeroneb Go^®^, 0.6 ± 0.0 and 0.6 ± 0.0 mg for salbutamol PMDI, 2.6 ± 0.2 and 1.5 ± 0.2 mg for terbutaline Turbuhaler^®^ DPI. The mass of particles smaller than 5 μm was also similar at ambient conditions and HPTH for all the devices except for terbutaline Turbuhaler^®^ DPI: it was, respectively, 2.0 ± 0.2 and 2.2 ± 0.2 mg for PariLC Star^®^, 2.9 ± 0.1 and 2.9 ± 0.2 mg for Aeroneb Go^®^, 15.8 ± 0.5 μg/puff and 15.8 ± 1.3 μg/puff for salbutamol PMDI, 82.8 ± 8.7 μg/puff and 51.9 ± 3.3 μg/puff for terbutaline Turbuhaler^®^ DPI. Figure 4 shows the deposited mass on impactor stages with the differents devices at HPTH and ambient air conditions.

**TABLE 2:** MMAD, total deposited mass on the stages of the ACI, % recovery from the impactor and the trachea of the nebulizer load for the nebulizers and of the emitted dose for the pMDI and DPI, and mass of particles smaller than 5 μm of terbutaline (5 mg/2 mL) nebulized with PariLC Star and Aeroneb Go^®^ nebulizers, salbutamol pMDI (100 μg/puff) and terbutaline Turbuhaler^®^ DPI (500 μg/puff) for both ambient conditions and 37°C-humidified (HPTH: human physiological temperature and humidity), expressed as mean ± standard deviation.

		**MMAD (μm)**	**Total deposited mass on the stages of the ACI (mg) (% of the nebulizer load or emitted dose)**	**% recovery from the trachea of the nebulizer load or emitted dose**	**Mass of particles < 5 μm**
PariLC Star (1) + terbutaline	Ambient T and RH	2.1 ± 0.3	2.3 ± 0.2 (43 ± 3%)	6 ± 3%	2.0 ± 0.2 (mg)
	HPTH	1.6 ± 0.1	2.5 ± 0.3 (47 ± 4%)	6 ± 3%	2.2 ± 0.2 (mg)
Aeroneb Go^®^ (2) + terbutaline	Ambient T and RH	3.3 ± 0.1	3.9 ± 0.3 (76 ± 1%)	6 ± 1%	2.9 ± 0.1 (mg)
	HPTH	2.0 ± 0.2	3.9 ± 0.3 (74 ± 5%)	10 ± 5%	2.9 ± 0.2 (mg)
Salbutamol pMDI (3)	Ambient T and RH	3.1 ± 0.0	0.6 ± 0.0 (26 ± 1%)	74 ± 1%	15.8 ± 0.5 (μg/puff)
	HPTH	3.1 ± 0.1	0.6 ± 0.0 (28 ± 2%)	72 ± 2%	15.8 ± 1.3 (μg/puff)
Terbutaline DPI (4)	Ambient T and RH	3.7 ± 0.1	2.6 ± 0.2 (41 ± 3%)	59 ± 3%	82.8 ± 8.7 (μg/puff)
	HPTH	3.2 ± 0.1	1.5 ± 0.2 (25 ± 3%)	75 ± 3%	51.9 ± 3.3 (μg/puff)

*(1) jet nebulizer, (2) vibrating mesh nebulizer, (3) presurized metered-dose inhaler (pMDI), (4) dry powder inhaler (DPI).*

## Discussion

This study developed an operational method of aerosol sizing at human humidity and temperature conditions and taken into account inhaled air at ambient temperature and humidity. This study has not the objective to be an alternative of regulatories/standard methods but to provide scientific information regarding the effect of temperature and humidity in inhaled condition on particle size and consequently deposition. Standard methods measure particle sizes emitted by the device. Our method has the objective to measure the particle size in the airways.

The first step of the study, consisting in sizing 1%w/v NaF aerosol nebulized with PariLC Star^®^ nebulizer for three intermediate experimental set-ups, highlighted the effect of temperature and humidity on particle size. It demonstrated that MMAD decreased significantly when heating to 37°C as the particles evaporated (2.6 vs. 1.3 μm); it then increased when RH was brought to saturation due to condensation, i.e., hygroscopic growth (1.3 vs. 1.8 μm). The second set-up may have been subjected to a decrease in relative humidity below ambient conditions, and thus conditions in the trachea and cascade impactor for this experimental set-up may have been drier than ambient conditions. The decrease of MMAD when comparing it at ambient temperature and RH and at HPTH (2.6 vs. 1.8 μm) leads to the conclusion that evaporation has more impact than condensation. This may be due to the functioning of the PariLC Star^®^ nebulizer: the air circulates through the interior of the PariLC Star^®^ and becomes saturated with humidity drawn from the large reservoir of solution (Nerbrink et al., 2003), so the air carrying the aerosol is already saturated when it comes out of the nebulizer. Prediction of lung deposition was consequently higher in the peripheral region at HPTH than at ambient conditions (MMAD smaller at HPTH than at ambiant condition), but both conditions (HPTH and ambient) predicted a significant deposition in the pulmonary region (mass of particles < 5 μm). These results could explain why Sangwan et al. (2003) have correlated cascade impaction data with deposition studies and found that a more meaningful cut off is 2.5 μm instead of 5 μm. Our work also tested the three main types of device: two nebulizers (a jet nebulizer and a vibrating mesh nebulizer), a PMDI and a DPI. This enabled the behavior of each kind of device to be studied for the two conditions of temperature and humidity (ambient and HPTH), and to determine their respective sensitivity to high temperature and humidity. The comparison of particle size distributions sized at ambient conditions and at HPTH showed that MMAD at HPTH was smaller than at ambient conditions for the two nebulizers (PariLC Star^®^ and Aeroneb Go^®^) with terbutaline, which is consistent with the results described above for NaF solution aerosolized with PariLC Star^®^. This indicates that particle evaporation was greater than hygroscopic growth.

An explanation of this low effect of condensation, even with the Aeroneb Go^®^ nebulizer, could be the same as that given by Finlay and Smaldone (1998), who emphasized that much of the understanding of hygroscopic aerosols is based on considering the fate of single droplets which may behave quite differently from the clouds of droplets produced by nebulizers, as it is possible for such clouds to behave in a non-hygroscopic way. Aerosols with large numbers of droplets per unit volume, such as can occur with nebulized aerosols, can actually self-humidify the air around them and thereby prematurely stop hygroscopic size changes. This phenomenon, known as the “two-way coupled effect,” occurs when each droplet shrinks only slightly, but the number of droplets is so great that the vapor evaporating off the droplets into the surrounding air causes the air to reach water vapor equilibrium (Finlay, 1998). Thus, the “two-way coupled effect” may stabilize some hygroscopic aerosols against size alteration in the respiratory tract. While the airflow rate of our model was fixed, changing the airflow rate may change the dilution of the aerosol in the air, which could give different results.

This result is consistent with the study of Martin et al. (2005) who observed no significant difference in the evaporation rate of PMDI between dry and humid conditions at 37°C, i.e., PMDI particles did not evaporate more slowly in the presence of high levels of humidity. In their study, the droplets placed at 37°C evaporated under both humid and dry conditions, showing that evaporation was greater than condensation. Another study performed by Martin and Finlay (2005) led to the hypothesis that in humid conditions, PMDI particles initially undergo a rapid evaporation of propellant from residual drug particles which quickly reduces aerosol diameters, then a transient growth of propellant-cooled particles due to condensation of water, followed by a water re-evaporation at a steady, warmer temperature. Thus, given sufficient time, quasi-steady state evaporation of water from PMDI particles may largely negate the initial condensation.

For the nebulizers and the salbutamol PMDI, there was no difference in total drug deposition on the ACI stages between ambient conditions and HPTH, indicating that particles did not grow at the entrance of the trachea (before being impacted on the impactor stages) at HPTH. However, the results obtained for terbutaline Turbuhaler^®^ DPI showed many fewer particles were deposited in the cascade impactor at HPTH than at ambient conditions, indicating that more particles were deposited in the trachea at HPTH. This would mean that at HPTH, particles grew very rapidly at the entrance of the trachea due to condensation, and impacted on the walls of the trachea and the cylinder. As a result, the mass of particles smaller than 5 μm at HPTH was nearly half that under ambient conditions. Terbutaline Turbuhaler^®^ DPI was the only device tested whose particles grew, i.e., underwent more condensation than evaporation. This phenomenon may be explained by the fact that terbutaline Turbuhaler^®^ DPI is a powder, and thus much more subject to hygroscopic growth than a solution. Moisture is well known to affect powder cohesion through capillary force at high relative humidity (Telko and Hickey, 2005; Chan, 2006).

There are clearly some limitations to this study. The flow chosen were constant and did not follow a pattern of breathing. The flow of 28.3 L/min was that recommended for the ACI and has stood the test of time with regard to accuracy. It is also a reasonable approximation of the mean inspiratory flow of an adult (tidal volume 750 mL and inspiratory time of 1.5 s). However, it would be too low to fully activate the DPI device so the flow of 60 L/min was chosen with a mathematical recalculation of the cut points which could have introduced some inaccuracies. This flow would be in the same order of magnitude as that expected from a patient inhaling forcefully from the device. These studies are in contrast to those of where Sangwan et al. (2003); Solomita and Smaldone (2009), Sagalla and Smaldone (2014), and Samuel and Smaldone (2020) used a low flow impactor to sample the aerosol rather than directing the entire output of the device into the impactor. This set up has the advantage to use a pattern of breathing but eliminated the “throat” where, in the present setup, it is expected that most of the changes in particle size occurred. NGI cascade impactor with a lower flow rate at 15 L/min is recommended for nebulizers particle size measurement (ISO 27427, 2012). Our method could be adapted with the NGI cascade impactor. A further issue is that they used either normal saline (Solomita and Smaldone, 2009; Sagalla and Smaldone, 2014) or γ interferon (Sangwan et al., 2003; Samuel and Smaldone, 2020) whereas the focus in the present study was asthma medication and the results suggest that changes in particle size due to exposure to HPTH conditions are specific for individual formulations. Finally, this was an *in vitro* study designed to evaluate temperature and humidity on various asthma medication and their delivery systems and does not have the power of *in vivo* deposition studies (Sangwan et al., 2003; Sagalla and Smaldone, 2014; Samuel and Smaldone, 2020) to predict pulmonary deposition in the face of significant disease.

The overall comparison of the four devices tested predicted a major deposition in the central and peripheral regions of the lung for the nebulizers (PariLC Star^®^ and Aeroneb Go^®^), and in upper airways for the salbutamol PMDI and the terbutaline Turbuhaler^®^ DPI. Thus PariLC Star^®^ and Aeroneb Go^®^ may allow the desired site of action to be targeted, i.e., the lung, whereas most of the drug delivered by salbutamol PMDI and terbutaline Turbuhaler^®^ DPI may be lost in the upper airways. The high injection speed for the salbutamol PMDI and the 60 L/min sampling flow rate for the terbutaline Turbuhaler^®^ DPI are mainly responsible for this large deposition in the trachea (Newman et al., 1996; Newman, 2003).

In this study, the nebulizers were associated with terbutaline solution. It is essential to consider the “nebulizer + solution to nebulize” couple and not the nebulizer alone, as one nebulizer can produce a different aerosol with different solutions. Results may have been different if another drug had been used. Formulation affects the particle growing/evaporation and consequently deposition prediction.

The potential change in cut-off diameters of the cascade impactor stages at 37°C was taken into account. Particle collection at the impactor stage is governed by the Stokes number. The cut-off diameter of the corresponding stage can be calculated from the Stokes number and depends on air viscosity. Air viscosity is defined by Willeke and Baron (1993), Baron and Willeke (2001):

$$\mu =\frac{\mu \,\left(r\,e\,f\right)*\left(T\,\left(r\,e\,f\right)+S\right)}{\text{T}+\text{S}}*{\left(\frac{\text{T}}{T\,\left(\mathrm{ref}\right)}\right)}^{3/2}$$

where μ(ref) is the reference air viscosity (183.25 micropoise), T(ref) is the reference temperature (293.15 Kelvin), S is the Sutherland constant (110.4 Kelvin).

When comparing air viscosity at 20°C (ambient temperature) and 37°C, a deviation of 4% was observed. This deviation is not significant compared to the bias between the experiments.

The Stokes number can be defined by Hinds (1999), Willeke and Baron (1993), Baron and Willeke (2001):

$$S\,t=\frac{\rho_{p}*d_{p}^{2}*Q*C_{p}}{9*\mu *W}$$

where ρ_*p*_ is the particle density (1,000 kg/m^3^), d_*p*_ is the particle diameter, Q is the jet velocity, C_*p*_ is the slip correction factor, μ is the air viscosity, W is the jet diameter.

For d_*p*_ = 3 μm and W = 0.0025 m, a deviation of 2% was observed when comparing air viscosity at 20°C (ambient temperature) and 37°C.

Mathematical models, which can be used to predict aerosol deposition in the respiratory tract, require knowledge of many parameters, including the characteristics of the drug, the aerosol generator and the aerosol itself. As the aerosol particle size has to be known, it seems more relevant to perform the sizing directly at HPTH to predict drug deposition. This is a considerable advantage of the method proposed in this study which allows study of the effect of temperature and humidity on the aerosol generated by the device and not on the device itself, a distinction which is not always clearly made. Moreover, our model is close to human physiological conditions where the air carrying the aerosol came from the ambient atmosphere, which is similar to the clinical setting. The continuous airflow may approximate the deep inhalation for PMDI and DPI, but did not simulate patient breathing for nebulizers. However, this study was an initial step in exploring the potential for hygroscopic growth and other changes to the aerosol that could take place while particle sizing with the ACI. The results obtained in this study for the nebulizers are consistent with the study of Fleming et al. (2006) which showed that alveolar deposition (i.e., small particles) was greater for *in vivo* experiments than for the LUDEP deposition modeling program. Our results could explain this difference. Finally, terbutaline Turbuhaler^®^ DPI was the only device subject to hygroscopic growth, since its mass of particles smaller than 5 μm decreased at HPTH, whereas it did not change for the nebulizers and the salbutamol pMDI.

## Conclusion

An aerosol sizing at controlled temperature and humidity with the respect of ambient air before aerosol delivery has been developed using a cascade impactor. Using physiological temperature (37°C) and humidity (100%RH) conditions vs. ambient air condition, we observed a decrease of particle size for liquid aerosol produced by nebulizers; no particle size change for PMDI, and a decrease of mass of particle smaller than 5 μm suggesting a rapid particle growing for powder aerosol produced by a DPI. Scintigraphic measurement obtained by Sangwan et al. (2003) support our results obtained with nebulization. *In vivo* deposition studies have to be conducted to evaluate the relevance of this method for aerosol deposition prediction.

## Data Availability Statement

The datasets presented in this study can be found in online repositories. The names of the repository/repositories and accession number(s) can be found in the article/supplementary material.

## Author Contributions

CM, AC, and LV conducted experiments and analyzed results. AC, AL, and LV provided scientific input and contributed to experiment design. CM and LV analyzed experiments and drafted the manuscript. AC, AL, and LV designed and coordinated the overall project. All authors contributed to the article and approved the submitted version.

## Conflict of Interest

LV has been employed by DTF Medical (Saint Etienne, France) from 2001 to 2018 and by Nemera (La Verpilliere, France) from 2018 to 2020. LV reports research support from Aerogen, Aptar Pharma, Astra Zeneca LFB, Nemera, Protec’som, and Erempharma. The remaining authors declare that the research was conducted in the absence of any commercial or financial relationships that could be construed as a potential conflict of interest.
